# Ultrafast momentum-resolved visualization of the interplay between phonon-mediated scattering and plasmons in graphite

**DOI:** 10.1126/sciadv.adu1001

**Published:** 2025-04-02

**Authors:** Francesco Barantani, Rémi Claude, Fadil Iyikanat, Ivan Madan, Alexey A. Sapozhnik, Michele Puppin, Bruce Weaver, Thomas LaGrange, F. Javier García de Abajo, Fabrizio Carbone

**Affiliations:** ^1^ Institute of Physics, École Polytechnique Fédérale de Lausanne, Lausanne, 1015, Switzerland.; ^2^ Department of Quantum Matter Physics, University of Geneva, Geneva, 1211, Switzerland.; ^3^ Lausanne Centre for Ultrafast Science, École Polytechnique Fédérale de Lausanne, Lausanne, 1015, Switzerland.; ^4^ ICFO-Institut de Ciencies Fotoniques, The Barcelona Institute of Science and Technology, Castelldefels (Barcelona), 08860, Spain.; ^5^ ICREA, Institució Catalana de Recerca i Estudis Avançats, Barcelona, 08010, Spain.

## Abstract

Scattering between charges and collective modes in materials governs phenomena such as electrical resistance, energy dissipation, and phase switching. Studying such scattering requires simultaneous access to ultrafast and momentum-resolved dynamics of single-particle and collective excitations, which remains an experimental challenge. Here, we present time- and momentum-resolved electron energy loss spectroscopy, and we apply it to graphite, demonstrating that large (Δ*q* ≃1.2 Å^−1^) photoexcited electron-hole pockets induce a renormalization of in-plane and bulk plasmons. This effect is explained by intra- and intervalley scattering processes mediated by E_2g_ and $A\prime_{1}$ phonon modes, which we directly observe via ultrafast electron diffraction and identify via ab initio calculations. Conversely, smaller electron-hole pockets (Δ*q* ≃0.7 Å^−1^) result in the renormalization of in-plane plasmons, which can only be partially explained by phonon-mediated scattering and thermal expansion. Our results highlight the importance of combining momentum- and time-resolved information to elucidate electronic scattering processes.

## Teaser

Experiments with time, energy, and momentum resolution track quasi-particles, revealing their microscopic interactions.

## INTRODUCTION

Understanding and harnessing the properties of quantum materials are key to implementing advanced functionalities in future devices. In the realm of two-dimensional (2D) materials, a vast range of engineered materials are accessible through approaches that include assembling layered heterostructures (*1*), controlling the interlayer twist angle (*2*), in-plane sliding (*3*), or exploiting the valley degree of freedom (*4*). The latter leverages on manipulating carriers present at specific valleys in the electronic band structure (i.e., minima of the conduction band or maxima of the valence band) close to the Fermi level (*5*). Graphene is a prototypical 2D system, presenting inequivalent Dirac points where valley polarization is observed (*6*) and thus serving as an ideal model for exploring and investigating valleytronic scenarios.

Most of the studies on valley polarization were performed by applying optical techniques and focused on emergent excitons with a specific valley character (*5*). Deeper insight into intervalley scattering can be obtained by gathering time-resolved information to extract, for example, specific relaxation timescales given by different scattering channels. However, because of the small momentum carried by visible photons, optical measurements are limited to the exploration of scattering processes involving collective modes close to the Γ point. This severely limits the possibility of studying processes at finite momenta, such as those mediated by zone-boundary phonons (*7*) or involving dark excitons (*8*).

In addition, finite momentum spectroscopies resolved on ultrafast timescales are mostly restricted to study the single-particle response. This is the case of time-resolved angle-resolved photoemission spectroscopy (tr-ARPES), where the effect of bosonic interactions is averaged over momentum transfers within the Brillouin zone (BZ). Only recently, x-ray free-electron lasers have enabled the realization of direct finite-momentum spectroscopy of the transient collective response in quantum materials (*9*–*11*), which complements the information acquired on the single-particle response.

**Fig. 1.**
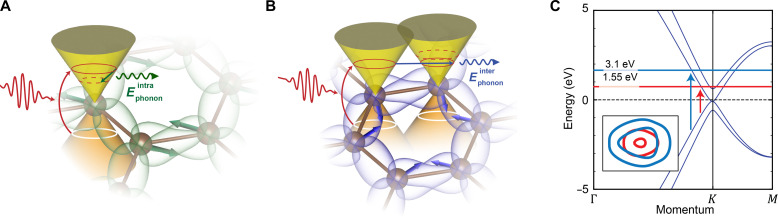
Carrier scattering in photoexcited graphite. Sketch of intravalley (**A**) and intervalley (**B**) scattering processes occurring in graphite. An ultrafast laser pulse photoexcites the electrons, which can relax through the so-called strongly coupled optical phonons. Atomic displacements are indicated by green and blue arrows for the E_2g_ (intravalley) and $A\prime_{1}$ (intervalley) phonon modes, respectively. To pictorially show the spatial distribution of electronic charges reacting to the above-mentioned scattering processes, we plot the charge density associated with molecular orbitals of the π bands crossing the Fermi level. The collective charge response is affected differently by intra- and intervalley scattering processes. (**C**) Band structure of graphite in the vicinity of the K point obtained using a tight binding model (*49*). We represent the 1.55- and 3.1-eV photoexcitations with red and blue arrows, respectively. The inset shows the photoexcited electron pockets resulting from the two photon energies.

Here, we use a combination of momentum-resolved techniques to investigate the ultrafast scattering processes occurring across different valleys in graphite. First, through high signal-to-noise-ratio ultrafast electron diffraction (UED) measurements, we obtain the out-of-equilibrium population of the phonons involved in the different scattering mechanisms ensuing laser photoexcitation. Subsequently, we carry out time- and momentum-resolved electron energy-loss spectroscopy (tr-q-EELS) in an ultrafast transmission electron microscope (UTEM) to visualize the impact of such intra- and intervalley scattering processes on collective electronic modes (plasmons). After photoexcitation close to the Dirac points, hot electrons induce an out-of-equilibrium phonon population (*12*, *13*), which produces scattering within the same valley—thus interacting with E_2g_ phonons close to the Γ point—or across different valleys—involving $A\prime_{1}$ phonons with momentum close to K. These two relaxation mechanisms are illustrated in Fig. 1 (A and B), respectively, together with a sketch of the charge density distribution in graphite. The population of these strongly coupled optical phonons modifies the charge distribution in graphite by distorting the structure along the phonon eigenvector displacements. This structural distortion alters Coulomb screening and induces a transient dielectric response in photoexcited graphite, leading to distinct effects on the two scattering processes.

In graphite, one can readily harness the excitation photon energy to tune the size of photoexcited electron-hole (e-h) pockets in the electronic momentum distribution. We use near-infrared (NIR; 1.55 eV) and visible (3.1 eV) pulses to respectively photoexcite small (Δq ≤ 0.7 Å^−1^) and large (Δq ≥ 1.2 Å^−1^, more isotropic) e-h pockets around the K points, as depicted in Fig. 1C. We then monitor the impact of phonon-mediated charge-carrier relaxation through intra- and intervalley scattering processes, first by measuring the ultrafast diffused diffraction intensity and secondly by tracking the evolution of plasmonic collective modes by tr-q-EELS.

The temporal evolution of both the in-plane π and the bulk σ + π plasmons that we observe for 3.1-eV photoexcitation can be quantitatively modeled by ab initio calculations, considering a large out-of-equilibrium population of E_2g_ phonons mediating the intervalley scattering processes. Diffuse scattering measurements confirm this scenario by revealing an intense signature of phonons near the Γ point, directly related to the electronic relaxation of large e-h photoexcited pockets. Instead, for the smaller NIR-excited pockets, we need to also consider the out-of-plane thermal expansion produced by an elevation in lattice temperature to qualitatively reproduce the experimental loss function dynamics, while we cannot fully explain the π plasmon dynamics.

The π plasmon, which in previous studies was buried in the elastic peak and so impossible to observe (*14*), undergoes different dynamics for the two pump energies. This observation can be rationalized by considering that the π plasmon results from the π and π^⋆^ bands that cross the Fermi surface and can experience additional screening due to the increases in phonon population and electronic temperature. Furthermore, previous studies (*15*) suggested that the same π and π^⋆^ bands can show a strong electronic renormalization when resonantly excited.

Because of its profound similarities with graphene, and thanks to its relatively well-understood behavior under equilibrium conditions, out-of-equilibrium graphite has been the subject of extensive investigations throughout the last decades. Optical (*12*, *13*, *15*, *16*), structural (*17*, *18*), and electronic measurements at high energies (*14*, *19*, *20*) provided insights into the characteristic relaxation channels on different timescales, while ultrafast diffused electron scattering (*7*, *21*) and tr-ARPES (*22*–*25*) experiments revealed the important role played by strongly coupled optical phonons in the scattering processes. The plasmonic properties of graphite have also attracted attention due to the long lifetimes observed in the low-energy plasmons of highly doped graphene and their potential for applications (*26*, *27*). However, direct observation of the interplay between single-particle excitations and collective electronic and structural modes is lacking to date due to the challenge involved in obtaining microscopic information that is simultaneously momentum and time resolved.

## RESULTS

**Fig. 2.**
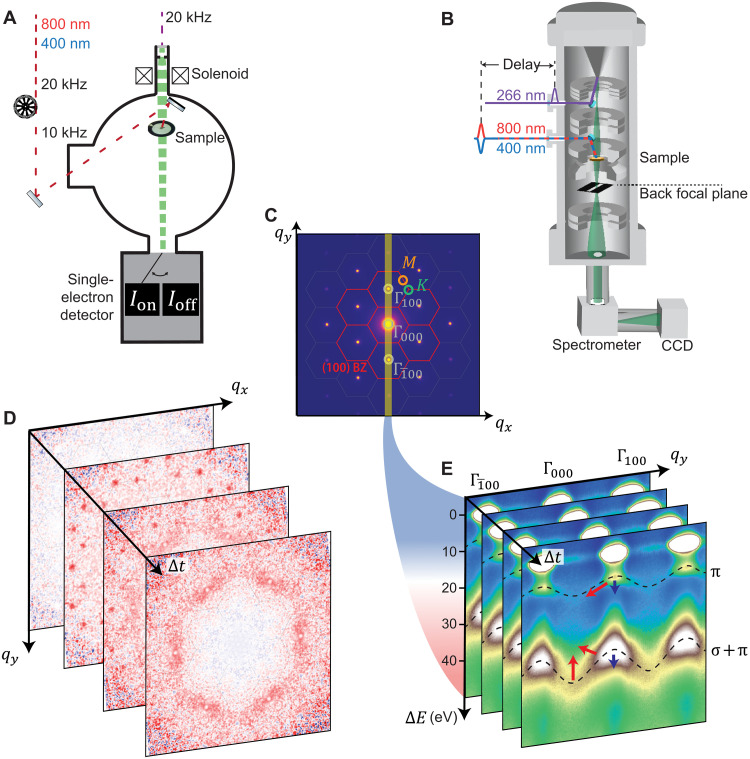
Illustration of the experimental approach used here for momentum-, time-, and energy-resolved measurements. (**A**) Scheme of the UED setup used to measure the diffraction patterns (C) resulting from the scattering of an electron pulse (green) after the photoexcitation by an optical pulse (red). (**B**) Scheme of the tr-q-EELS experiment: An electron spectrometer analyzes the energy of the scattered electrons selected by a slit oriented along the Γ → M direction [orange rectangle in (C)]. (**C**) Diffraction pattern of graphite with hexagons indicating the BZ. The (100) BZ family is highlighted in red, among which Γ_100_ and $\Gamma_{\bar{1}00}$ are Bragg spots highlighted in gray, together with the unscattered beam Γ_000_. High-symmetry points (M and K) are indicated in orange and green, respectively. (**D**) Examples of transient diffraction patterns captured with UED for four different time delays Δ*t*. (**E**) Electron energy loss spectra at different time delays Δ*t* resolved in the scattering vector *q*_*y*_. The dashed curves are guidelines for the π and π + σ dispersion curves. Arrows show the trends of the plasmon dynamics with 1.55- (red) and 3.1-eV (blue) photoexcitations. CCD, charge-coupled device.

Figure 2 illustrates our combined experimental approaches: UED in Fig. 2A and tr-q-EELS in Fig. 2B, both of which involve an electron pulse that is transmitted through a graphite flake excited by an ultrafast laser pulse. UED provides access to the response in reciprocal space: incoherent lattice motion, Debye-Waller effect, and phonon population imprinted on the Bragg and diffused electrons, allowing for the measurement of the time-resolved dynamics of low-energy collective modes, as represented in Fig. 2D. Tr-q-EELS tracks the spectroscopic response of the inelastically scattered electrons, yielding the energy and momentum-resolved loss function of the material. In this study, we place a slit in reciprocal space to select a specific momentum region oriented along the desired direction, and we collect energy-loss spectra using an electron spectrometer combined with our UTEM. This results in a direct measurement of the ultrafast electronic collective mode dispersion as a function of transferred momentum, illustrated in Fig. 2E.

### Ultrafast electron diffraction

We present UED measurements performed on a 50-nm-thick natural graphite flake with a shot-to-shot single electron detector acquisition (*28*). To visualize the photoinduced signal, we normalize the diffraction patterns by dividing those acquired with pump excitation by those acquired without it and then subtracting the same ratio computed from the averaged patterns before excitation, as described by Eq. 1.

**Fig. 3.**
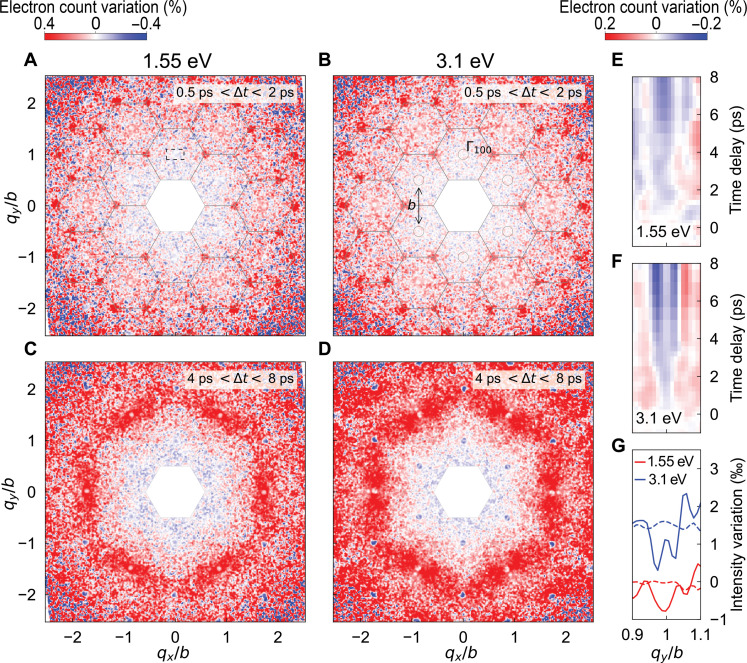
Ultrafast diffuse response measured by UED. (**A** to **D**) UED maps of graphite after excitation normalized to the unpumped diffraction patterns with 1.55-eV [(A) and (C)] and 3.1-eV [(B) and (D)] photon energy. (A) and (B) are averaged over early time delays (0.5 to 2 ps), while (C) and (D) are averaged over later time delays (4 to 8 ps). (**E** and **F**) Normalized diffraction patterns binned along the Γ → M direction [within the dashed rectangle in (A)] as a function of time delay after the photoexcitation by 1.55- and 3.1-eV pump pulses, respectively. (**G**) Line cuts of (E) and (F) for 1.55- and 3.1-eV pump energy, respectively. Solid lines are averaged between 4 and 8 ps, and dashed lines are taken before time zero. The color bar on the top left corresponds to the color scale in (A) to (D) and the color bar on the top right to (E) and (F). The wave vector is normalized to the reciprocal lattice unit *b* = 2.95 Å^−1^.

Figure 3 (A to D) shows the diffused scattering response averaged over two different time-delay intervals (0.5 to 2 ps and 4 to 8 ps) for the two pump energies (1.5 and 3.1 eV) at the same absorbed fluence of 0.8 mJ/cm^2^. Each Bragg peak corresponds to the center of each BZ, represented by hexagons. At early stages (0.5- to 2-ps time delay), we observe a strong intensity increase at the K points of the BZ (Fig. 3, A and B)—an effect produced by the increase in the population of $A\prime_{1}$ phonons (*7*). At later delays (4 to 8 ps), we observe an increase in intensity between the second-order Bragg peaks and the M points of the reduced BZ, which we attribute to excitation of transversal acoustic phonons with a momentum spanning the Γ → M direction. Because of the low excitation fluence, the incoherent thermal motion of the lattice is small as confirmed by the weak Debye-Waller effect at the Bragg peak (see fig. S4A).

To study the behavior of the Γ_100_ Bragg peak, we integrate the diffraction patterns along the *q*_*x*_ axis (in the region illustrated by the dashed rectangle in Fig. 3A) and plot the dynamics along the Γ → M direction. The results are represented in Fig. 3 (E and F), where we observe a decrease in intensity at the Bragg peak positions for both photoexcitation energies. We focus on the signal between 4 and 8 ps after the photoexcitation for the two pump energies, whose time-delay average is shown in Fig. 3G. While the Γ_100_ peak decreases in intensity for 1.55-eV excitation, with 3.1 eV, the peak also becomes more narrow (see fig. S5). This suggests an increase in phonon population at small momenta (i.e., E_2g_ phonons) when graphite is irradiated with pulses of higher photon energy and similar fluence. In summary, we find that the (100) Bragg peak exhibits a remarkable difference along the Γ → M direction between the two photon energies, and therefore, we perform tr-q-EELS experiments along that direction to investigate whether and how the low-energy phonon modes affect the high-energy electronic response.

### Time- and momentum-resolved EELS: Experiments and simulations

Previous investigations of graphite by ultrafast EELS were limited to the bulk σ + π plasmon dynamics (*14*) or focused on core-loss peaks to extract the evolution of the local density of states (*20*). Our approach overcomes such limitations and allows us to also observe the π plasmon, located at around 7 eV and directly originating from interband transitions between the π and π* electronic bands. As depicted in Fig. 2, we identify the π plasmon in the EELS spectra at low energies and the σ + π plasmon at around 30 eV. Following the dispersion of both plasmons up to the zone boundary, we notice a typical quadratic dispersion consistent with previous literature (*29*). However, the limited energy resolution of our ultrafast electron microscope does not allow us to directly resolve phonon losses, which are buried within the zero-loss peak. In the Supplementary Materials, we discuss other time-resolved contributions to the EELS response that are not due to photoexcited carrier scattering.

In the dynamics captured by tr-q-EELS, we expect the π plasmon to be strongly influenced by the laser photoexcitation and the subsequent scattering processes. On the other hand, the σ + π plasmon at higher energies is known to be strongly affected by the interlayer spacing (*30*). In our experiments, we illuminate a flake of highly ordered pyrolytic graphite (HOPG) oriented along the [001] zone-axis in analogous conditions to the UED measurements, with 1.55- and 3.1-eV ultrafast pulses. We compare the variation of the EELS spectra as a function of momentum, along the Γ → M high-symmetry direction as motivated before. In the analysis of the tr-q-EELS experiments, we integrate the spectra before excitation by optical pulses to represent the equilibrium situation, and the spectra for relatively long time delays averaged between 2 and 8 ps to study the photoexcited specimen. In this way, we avoid both spurious effects from interaction with near-fields (see the Supplementary Materials for details) and fast out-of-equilibrium electronic contributions. We further normalize the spectra in the energy 5- to 50-eV range as a function of the transferred momentum and plot the observed variation relative to the equilibrium response.

**Fig. 4.**
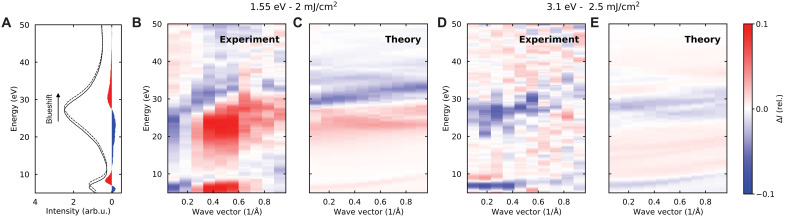
Experimental and theoretical variation of the EELS response in photoexcited graphite. (**A**) Graphite EELS response at the Γ_000_ point. We show a spectrum acquired from unperturbed graphite (solid curve), a blue-shifted version of this spectrum (dashed curve), and the difference between them (filled blue/red areas for negative/positive values). (**B** to **E**) Wave vector dependence of the pump-induced change in EELS. [(B) and (D)] Maps measured along the Γ → M direction for 1.55-eV pump with fluence (2 mJ/cm^2^) (B) and 3.1-eV pump with fluence (2.5 mJ/cm^2^) (D). (B) and (D) are averaged between 2 and 8 ps. [(C) and (E)] Simulated maps under the conditions of (B) and (D), respectively (see the Supplementary Materials for details including the temperatures used in the simulations which are reported in table S2.). The experimental maps have been smoothed using a Savitzky-Golay filter along the energy axis.

Figure 4 compares the variation in the loss function at the two different excitation energies (1.55 and 3.1 eV). As discussed above, the size of the photoexcited pockets changes as a function of photon energy: The 3.1 eV-pump excitation induces larger e-h pockets in momentum space and, thus, requires phonons with smaller momentum *q* for intervalley scattering, leading to a more isotropic phonon distribution in momentum space. The energy-momentum maps in Fig. 4 (B to E) represent experimental and theoretical pump-induced EELS changes as a function of momentum and energy transfer, showing regions of alternating sign that can be attributed to plasmon shifts. To visualize this effect, we plot in Fig. 4A a characteristic EELS spectrum for undeflected electrons (i.e., at the Γ_000_ point) acquired from unperturbed graphite, where we observe two peaks corresponding to the in-plane and bulk plasmons described above; we then assume an excited spectrum with both plasmons shifted to higher energy and compute the difference with respect to the unshifted spectrum. The energy shift results in positive (red) and negative (blue) differential signals forming a pattern analogous to those in Fig. 4 (B to E) as a function of momentum.

When using NIR pulses (1.55 eV; Fig. 4B), we find that close to the Γ point, the intensity of the in-plane π plasmon decreases, and the bulk σ + π plasmon moves toward higher energies. We observe the opposite trend at larger momentum transfer, with an increase of the π plasmon and a more pronounced redshift of the bulk plasmon. The EELS response for 3.1-eV photoexcitation, illustrated in Fig. 4D, instead shows a different behavior, namely, the π plasmon shifts toward higher energies at small momenta (*q* ≤ 0.5 Å^−1^), while the bulk plasmon intensity of the σ + π peak decreases, indicating a broadening without any clear energy shift.

To understand the microscopic origin of the EELS response in out-of-equilibrium graphite, we perform ab initio simulations for both equilibrium and photoexcitation conditions. We identify two contributions to the excited state: the effect of thermal expansion—as the plasmonic response largely depends on the interlayer spacing (*30*)—and the variation in the population of strongly coupled optical phonons. For the former, we use in-plane and out-of-plane thermal expansion coefficients taken from the literature (*31*, *32*), which directly modify the lattice constants depending on an effective temperature *T*_L_, and this in turn results in a modified EELS probability ${\text{EELS}}_{L}\left(T_{L}\right)$. For optical-phonon coupling, we simulate the effect of a population of optical E_2g_ and $A\prime_{1}$ phonons at the Γ and the K points, respectively; more precisely, we calculate EELS spectra for distorted unit cells corresponding to different atomic displacements along these modes; the EELS probabilities ${\text{EELS}}_{A\prime_{1}}\left(T_{A\prime_{1}}\right)$ and ${\text{EELS}}_{E_{2g}}\left(T_{E_{2g}}\right)$ are then computed as the average weighted by the probability densities associated with each specific value of the displacement (i.e., the amplitude distribution associated to ladder modes in a harmonic oscillator, weighted by the Bose-Einstein distributions at the phonon temperatures $T_{A\prime_{1}}$ and $T_{E_{2g}}$, respectively). The simulated EELS probability is lastly obtained as ${\text{EELS}}_{\text{sim}}=w_{A\prime_{1}}{\text{EELS}}_{A\prime_{1}}\left(T_{A\prime_{1}}\right)+w_{E_{2g}}{\text{EELS}}_{E_{2g}}\left(T_{E_{2g}}\right)+w_{L}{\text{EELS}}_{L}\left(T_{L}\right)-{\text{EELS}}_{\text{eq}}$, with weights $w_{A\prime_{1}}=w_{E_{2g}}=0.25$ and $w_{L}=0.5$. We refer to Materials and Methods and the Supplementary Materials for further details, along with values of the fitted temperatures $T_{A\prime_{1}}$, $T_{E_{2g}}$, and *T*_L_ at each of the two pumping photon energies (see table S2).

The results of our simulations are shown in Fig. 4 (C and E), normalized as a function of momentum analogously to the experimental ones. For 3.1-eV pumping, we observe a large degree of similarity between the experimental and simulated maps. In particular, the experimental dynamics of both plasmons are well reproduced from small momentum up to *q* ≃ 1 Å^−1^ by considering out-of-equilibrium populations of the strongly coupled optical phonons. In addition, the experimental amplitude variation matches the simulations quantitatively. However, for NIR excitation, our simulations can only partially replicate the experimental observations. While the response at small momenta can be understood as arising from phonon contributions, the large variations of the plasmon at higher wave vector can only be reproduced by introducing an expansion of the interlayer spacing (the lattice term, *T*_L_, in the EELS simulations).

We can rationalize this by noting that structural distortions caused by excited phonons and lattice expansion lead to anisotropic modulations of interatomic distances, which in turn strongly affect the electronic band structure and Coulomb screening. Because of the difference in plasmon and phonon lifetimes ($\tau_{\text{phonon}}\gg \tau_{\text{plasmon}}$), no coherent plasmon-phonon coupling occurs. However, the plasmonic response—governed by charge distribution—is influenced by phonon-induced modifications, resulting in variations in plasmon behavior.

The discrepancy between theory and experiment under NIR pump excitation at small wave vector (e.g., *q* ≤ 0.3 Å^−1^) suggests that the pump induces long-range charge interactions. These interactions can break lattice symmetry and shift phonon frequencies, contributing to the observed changes in the plasmonic response. However, our first-principles calculations do not account for long-range charge modifications and therefore cannot reproduce experimental observations at low momentum transfer under NIR excitation.

In summary, thermal expansion along the vertical direction alters electron density, typically leading to plasmon redshifts, while phonon-mode excitations introduce an incoherent temporal averaging of the band structure, resulting in more intricate variations in plasmon peaks in the EELS spectra.

## DISCUSSION

Collective modes in materials emerge from the coherent excitation of structural, electronic, or magnetic degrees of freedom. They span a wide range of energies extending from micro–electronvolts in spin waves to milli–electronvolts in phonons or even to electronvolts in plasmons and excitons. In addition, these modes can couple together via specific interaction processes, giving rise to exotic many-body objects such as charged phonons (*33*) or phonon-assisted bimagnons (*34*, *35*). The capability to investigate the microscopic details associated with the interaction among collective modes and with single-particle excitations in materials is key to understanding the scattering mechanisms that govern the energy dissipation ensuing an out-of-equilibrium excitation and, more generally, the dynamics associated with energy pathways in excited materials. To this end, we have shown that one needs to combine spectroscopic and momentum-resolved information on the collective mode dynamics.

Ultrafast momentum-resolved EELS in a UTEM is a particularly powerful approach, as it grants continuous access to several momentum channels at once, in contrast to x-ray scattering or Raman spectroscopy, which can only measure specific momentum transfers, while being able to monitor a broad spectral range encompassing several electronic excitations of interest. Moreover, its combination with high signal-to-noise ratio UED complements this rich information by directly tracking across momentum space the diffuse scattering dynamics of low energy collective modes, which are not observable by tr-q-EELS because of its limited energy resolution.

In graphite, the photoexcited e-h pockets define the resulting inter- and intravalley scattering dynamics. Merging together the results from UED and tr-q-EELS, we observe that for different excitation energies, the preferential scattering channel varies and that the out-of-equilibrium phonon population renormalizes collective oscillations of the charge distribution. Our theoretical model accounts for the interactions between plasmons and phonons. Namely, we can quantitatively understand the response after a visible laser pulse, but our simulations fall short on explaining the plasmon dynamics with NIR photoexcitation. On the basis of current theoretical knowledge, we can only speculate on the causes of such an evident shift in plasmon energy that cannot be accounted for by phonon-mediated scattering. In our model, we did not consider the role of the high electronic temperature that can contribute to changes in the plasmon response (*22*).

We demonstrate that our comprehensive approach enables the resolution of microscopic details of collective modes in condensed materials, revealing that the scattering mechanism of charge carriers can be controlled by tuning the pump energy, resulting in the steering of the collective plasmonic response as a function of time and momentum on picosecond timescales. Leveraging the high spatial resolution of transmission electron microscopes, this method introduces an innovative tool for directly investigating the properties of emerging quasi-particles within nanoscale 2D platforms. Simultaneously, this protocol can be applied to other materials such as cuprates to unfold the interplay between high- and low-energy collectives modes (*36*–*38*) across the BZ. More generally, revealing the momentum dependence of collective excitations in photo-induced exotic phases (*39*–*41*) can help to shed light on their origin and unveil microscopic interactions in out-of-equilibrium strongly correlated states of matter.

## MATERIALS AND METHODS

### Sample preparation

Graphite flakes are obtained by mechanical exfoliation and deposited onto transmission electron microscopy copper grids once a thickness of approximately 50 nm is reached. For tr-q-EELS, we prepared flakes from HOPG and measured regions with typical single-crystal diffraction patterns, isolating them by inserting a selected-area aperture. For the UED measurements, we used natural graphite and selected an optically semitransparent flake with lateral dimensions of 500 by 300 μm^2^.

### Ultrafast electron diffraction

We perform UED measurements using a home-built tabletop setup, as described in (*28*). A Ti:Sapphire laser delivers 800-nm pulses with a 50-fs duration at a 20-kHz repetition rate. The third harmonic of the laser back-illuminates a silver-coated sapphire plate, generating nearly a thousand electrons per pulse, which are accelerated to 40 keV. At the sample position, the electron pulse duration is below 1 ps, with a beam waist of approximately 400 μm.

The pump beam’s repetition rate is halved to 10 kHz by a mechanical chopper, ensuring that the sample is photoexcited once every two probe pulses. Pump pulses at 1.55 eV (800 nm) and 3.1 eV (400 nm) uniformly excite a 400 × 400 μm^2^ region of the graphite flake. The acquisition system is phase-locked to the laser source, and a single-electron detector records each probe pulse. Two distinct diffraction patterns are alternately captured on separate chips: one in the presence of the pump, $I_{\text{on}}\left(q,t\right)$, and one without, $I_{\text{off}}\left(q,t\right)$, where *t* is the time delay and **q** is the scattering vector. After merging the intensity maps with the same delay and neutralizing pixels with outlier spikes, we average the diffraction patterns along the sixfold symmetry axis. To visualize the temporal variation of the scattered electrons in reciprocal space, we normalize the diffraction patterns as

$$\Delta I\left(q,t\right)=\frac{I_{\text{on}}\left(q,t\right)}{I_{\text{off}}\left(q,t\right)}-\frac{{\langle I_{\text{on}}\left(q,t\right)\rangle }_{t<0}}{{\langle I_{\text{off}}\left(q,t\right)\rangle }_{t<0}}$$ (1)

We report more details on the UED in the Supplementary Materials.

### Time- and momentum-resolved EELS

We perform tr-q-EELS measurements using a modified JEOL 2100 transmission electron microscope (TEM) operated at 200 kV (*42*, *43*), combined with a Ti:Sapphire amplified laser (Wyvern X, KMLabs) running at 1 MHz. Probe electrons are photoemitted by illuminating a LaB_6_ cathode with ultraviolet ultrashort pulses (4.65 eV, 267 nm), generated via third-harmonic generation from 800-nm pulses. The TEM is equipped with an electron spectrometer and a direct electron detector (Gatan K2), and energy-resolved spectra are acquired using a Gatan imaging filter system with a 0.05-eV-per-channel dispersion.

To isolate a specific momentum direction, we use a microfabricated aperture (*q*-slit) placed in the diffraction plane of the TEM. The selected diffraction pattern is imaged onto the spectrometer entrance, producing a 2D energy-momentum map. Graphite flakes, exfoliated onto a TEM Cu grid, are aligned with the *q*-slit using a double-tilt holder with an in-plane rotation stage. Out-of-equilibrium dynamics are triggered with pump pulses at 1.55 or 3.1 eV, focused to a spot with a diameter of 40 μm. Multiple maps, with a typical exposure time of 20 to 30 min, are acquired at different time delays to capture the dynamics.

### First-principles modeling of EELS in graphite

The electronic structure of graphite is obtained from first principles using density functional theory simulations with the Perdew-Burke-Ernzerhof exchange-correlation functional, as implemented in the Quantum Espresso code (*44*, *45*). Using the resulting Kohn-Sham wave functions and eigenenergies, we compute the macroscopic dielectric function within the independent-particle approximation using the YAMBO code (*46*). To determine the EELS probability, we calculate the in-plane $\epsilon_{∥}\left(q_{∥},\omega \right)$ and out-of-plane $\epsilon_{z}\left(\omega \right)$ components of the dielectric tensor of graphite, for in-plane wave vector transfer $q_{∥}$ and optical frequency ω. The EELS intensity, as a function of in-plane wave vector transfer and frequency, is then obtained using the following expression (*47*)

$$\Gamma_{\text{EELS}}\left(q_{∥},\omega \right)=\frac{e^{2}L}{\pi^{2}\hbar v^{2}}\text{Im}\left\{\frac{v^{2}/c^{2}-1/\epsilon_{∥}\left(q_{∥},\omega \right)}{q_{∥}^{2}+\left[\epsilon_{z}\left(\omega \right)/\epsilon_{∥}(q_{∥},\omega )\right]\left(\omega^{2}/v^{2}\right)-\epsilon_{z}\left(\omega \right)\omega^{2}/c^{2}}\right\}$$ (2)

here, *e* is the electron charge, *L* is the length of the electron trajectory inside the bulk of graphite, *v* is the electron velocity, and *c* is the speed of light.

To account for experimental observations, we simulate variations in the EELS probability due to atomic vibrations corresponding to the E_2g_ and $A\prime_{1}$ optical phonon modes, as well as thermal lattice expansion.

For phonon-induced variations, we estimate the change in the EELS probability by computing the permittivity for different bond displacements *x* around the equilibrium C─C bond distance *d*. The resulting EELS probability $\Gamma_{d+x}\left(q_{∥},\omega \right)$ depends on *x*. The thermally averaged EELS probability is then given by

$$\Gamma^{T}\left(q_{∥},\omega \right)=\int \mathrm{dx}\;\Gamma_{d+x}\left(q_{∥},\omega \right)P\left(x,T\right)$$ (3)

where P(x,T) is the temperature-dependent probability distribution for displacement *x*, obtained from a 1D quantum harmonic oscillator model.

To account for thermal expansion, we incorporate temperature-induced changes in interatomic bond distances and interlayer separations. Specifically, we use the in-plane, $\alpha_{a}\left(T\right)$, and out-of-plane, $\alpha_{c}\left(T\right)$, linear thermal expansion coefficients reported in (*32*), where $\alpha \left(T\right)=L^{-1}\left(dL/dT\right)$ and L represents the specimen length along the relevant crystallographic direction (*a* or *c*). By linearly interpolating these coefficients, we obtain the expanded lattice parameters of graphite at *T*_L_ = 600, 1000, 1500, and 2000 K. We then compute EELS maps for the corresponding atomic configurations.

To illustrate the effect of lattice expansion or the E_2g_ and $A\prime_{1}$ phonon modes on the graphite EELS probability, we calculate the temperature-induced variations in the EELS maps relative to room temperature, as described by $\Delta \Gamma \left(q_{∥},\omega \right)=\Gamma^{T}\left(q_{∥},\omega \right)-\Gamma^{\mathrm{RT}}\left(q_{∥},\omega \right)$. For a more detailed explanation of the ab initio calculations, see section S3 in the Supplementary Materials.

## Supplementary Materials

### The PDF file includes:

Sections S1 to S3 Figs. S1 to S9 Tables S1 and S2 Legend for movie S1 References
