# Supplementary material for: Ultrafast momentum-resolved visualization of the interplay between phonon-mediated scattering and plasmons in graphite
Source: Sci Adv. 2025 Apr 2;11(14):eadu1001. doi: 10.1126/sciadv.adu1001 (PMC11963994; doi:10.1126/sciadv.adu1001)
Supplement: Supplementary file 1 — Sections S1 to S3 Figs. S1 to S9 Tables S1 and S2 Legend for movie S1 References [file sciadv.adu1001_sm.pdf]

Science Advances
AAAS

Supplementary Materials for

Ultrafast momentum-resolved visualization of the interplay between phonon-mediated scattering and plasmons in graphite

Francesco Barantani *et al.*

Corresponding author: Fabrizio Carbone, fabrizio.carbone@epfl.ch

Sci. Adv. **11**, eadu1001 (2025)
DOI: 10.1126/sciadv.adu1001

The PDF file includes:

Sections S1 to S3
Figs. S1 to S9
Tables S1 and S2
Legend for movie S1
References

Other Supplementary Material for this manuscript includes the following:

Movie S1

S1. Experimental methods

A. Time- and momentum-resolved EELS

We perform time- and momentum-resolved electron energy-loss spectroscopy (tr-q-EELS) measurements in a modified JEOL 2100 transmission electron microscope (TEM) operating at an acceleration voltage of 200 kV (43, 44). Figure S1a shows the scheme of the experimental setup. The probe electrons are photoemitted by illuminating a truncated LaB_6 cathode with UV ultrashort pulses ($\simeq$ 4.65 eV, 267 nm), obtained by third-harmonic generation from the fundamental 800 nm pulses.

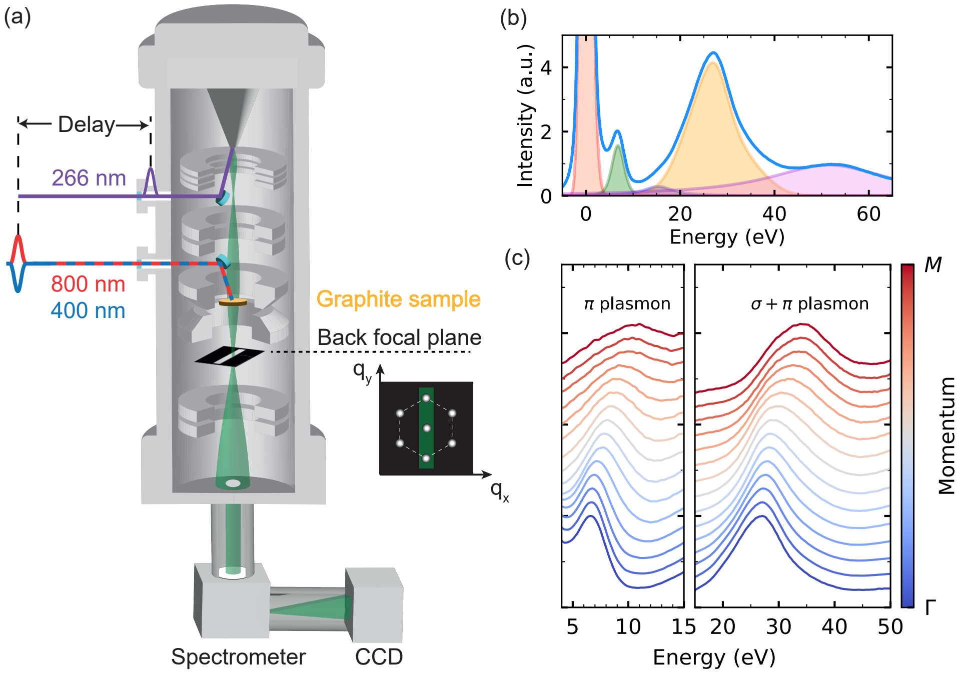

FIG. S1. **Illustration of the tr-q-EELS measurements of graphite.** (a) Experimental setup: ultrafast transmission electron microscope with a slit in the back focal plane to select electrons with a given in-plane momentum. (b) Typical electron energy-loss spectrum of graphite. The shaded areas indicate the plasmon peaks. c) Dispersion of the π and $\sigma + \pi$ plasmons. The colors correspond to the in-plane momentum transfer q (see color-coordinated scale).

Our transmission electron microscope is equipped with an electron spectrometer and a direct electron detector (Gatan K2). Energy-resolved spectra are recorded using a Gatan Imaging Filter (GIF) system operated with a 0.05 eV-per-channel dispersion setting. We

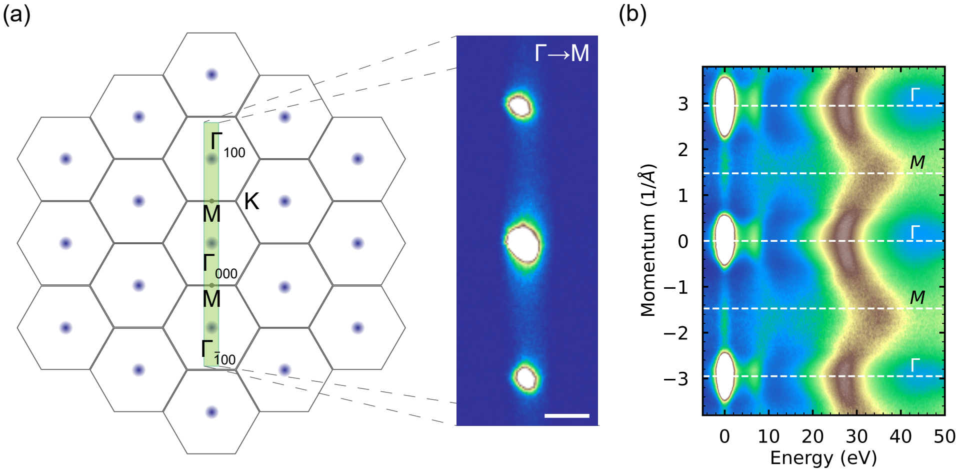

FIG. S2. **Image of the diffraction pattern selected by the slit and analyzed by the spectrometer.** (a) Momentum-space direction and Bragg spots selected by a q-slit with a real space image at the aperture of the GIF entrance. The white bar in the right color plot corresponds to 1 Å^{-1}. (b) EELS map normalized to the intensity in the 20–40 eV region.

use a typical exposure time of the direct CMOS sensor from 20 to 30 minutes.

To implement tr-q-EELS, we place a rectangular micro-fabricated aperture in the diffraction (back-focal) plane of the TEM, aligned along a specific momentum direction as shown in Fig. S2a. Consequently, the custom-made aperture acts at the focal plane where the diffraction pattern is formed. Next, we image the slit-selected pattern onto the spectrometer entrance aperture with the energy dispersion direction orthogonal to the momentum axis, thus obtaining a 2D energy-momentum map as shown in Fig. S2b. Finally, we acquire different maps at different time delays to retrieve the out-of-equilibrium dynamics.
The spectra shown in Fig. 4 of the main paper were smoothed using a Savitzky-Golay filter applied along the energy axis, with a window size of 31 points and a third-order polynomial interpolation.

The graphite flake is obtained by mechanical scotch-tape exfoliation and deposited on a TEM copper grid once a thickness of $\simeq$ 50 nm is reached. The alignment between the graphite pattern and the q-slit is done by an in-plane double-tilt holder: we could adjust the in-plane azimuthal angle for the desired direction in momentum space, and the two tilt angles to align the sample along the [001] zone axis. Given the thickness of the sample, we expect not to have contributions from multiple scattering processes, that would appear as a

shoulder above the bulk plasmon (50).
A typical EELS spectrum obtained on graphite is shown in Fig. S1b, with its dispersion as a function of momentum transfer represented in Fig. S1c.

To trigger out-of-equilibrium dynamics in graphite, we employ pump pulses with a central photon energy of either 1.55 eV (800 nm, near-infrared) or its frequency double 3.1 eV (400 nm, visible). The second-harmonic generation of the fundamental laser is done using a BBO crystal. We use a repetition rate of 1 MHz, which allows us to acquire enough statistics while making sure that the sample reaches thermal equilibrium between two pulses. The pump laser is focused on a spot of $\simeq 40$ μm diameter. We use Eq. (S2) to compute the absorbed fluence.

Most of the previous time-resolved EELS studies related to the photon-induced near-field electron microscopy (PINEM) technique (52), which involves laser and electron pulses arriving together at the sample so the latter interacts with the optical near-field induced near material structures. The electron spectrum broadens and exhibits energy sidebands equispaced by the photon energy. Even if we adjust the light polarization to minimize such a PINEM effect in our studies, it still emerges in the EELS maps. The very fast dynamics (time delay ≤ 500 fs) is therefore polluted by PINEM interactions in our study, and consequently, we discard it in our discussion.

In our experiments, we employed a photon energy able to excite electron-hole pairs around the K point - which occurs in the range from several hundred meV to around 2 eV. To be consistent with several previous investigations (7, 18), we chose 1.55 eV, which is the fundamental photon energy of the laser. We also took the second-harmonic wavelength to be as close as possible to the van Hove singularity in the band structure where the Fermi surface of the excited carriers changes topology. This happens at around 4.5 eV (16), where photoemission would also pollute the observed signal. Therefore, we decided to use a photon energy of around 3 eV, which was also convenient as it is the second-harmonic frequency of our laser.

B. Ultrafast electron diffraction

We perform ultrafast electron diffraction measurements in a home-build table-top setup described in Ref. (29), modified from Ref. (51), and represented in Fig. S3a. A Ti:Sapphire laser source provides ultrafast 800 nm pulses at a repetition rate of 20 kHz, with 50 fs pulse duration. The beam is stabilized with two motorized mirrors to avoid drifting. The third harmonic is used to back-illuminate a 40 nm silver coating on a sapphire plate and generate around 1000 electrons per pulse that are accelerated to 40 keV. The electron pulse duration is below 1 ps at the sample position with a beam waist of around 400 μm. The pump beam repetition rate is lowered to 10 kHz with a mechanical chopper such that the sample is photoexcited once every two probe pulses. The pump pulses at the two-photon wavelengths (400 and 800 nm) uniformly photoexcite a region of the graphite flake of 400×400 μm^2, corresponding to the full width at half maximum (FWHM) of the Gaussian beam in both directions. The acquisition system is phase-locked to the laser source. A single-electron detector acquires each probe pulse and saves alternatively the diffraction pattern onto two chips. We obtain two distinct diffraction patterns, one in the presence of the pump, $I_{\mathrm{on}}(\boldsymbol{q}, t)$, and another one without the pump, $I_{\mathrm{off}}(\boldsymbol{q}, t)$. The normalization, for which we use the expression

$$\mathit{\Delta} I(\boldsymbol{q}, t) = \frac{I_{\mathrm{on}}(\boldsymbol{q}, t)}{I_{\mathrm{off}}(\boldsymbol{q}, t)} - \frac{\langle I_{\mathrm{on}}(\boldsymbol{q}, t)\rangle_{t<0}}{\langle I_{\mathrm{off}}(\boldsymbol{q}, t)\rangle_{t<0}}, \tag{S1}$$

reveals the photoinduced scattered electron variation in the diffraction patterns along the time delay between the pump and probe pulses. This normalization is an effective way to perform gain referencing which reduces the noise.

The graphite sample was prepared the same way as described in Section S1 A. An optically semi-transparent flake of around 50 nm thickness and 500×300 μm^2 lateral dimensions was selected for the time-resolved measurements.

The pump power was set to match the same absorbed fluence for both pulse wavelengths. The absorbed fluence F_{abs} is defined as

$$F_{\mathrm{abs}} = \frac{P}{f \pi \sigma^2}(1 - R)A, \tag{S2}$$

where P is the measured power after the mechanical chopper, $f = 10$ kHz is the pump pulse repetition rate, σ corresponds to half of the FWHM, $R = \left(\frac{n-1}{n+1}\right)^2$ is the reflectivity and

λ (nm)	n	α (nm^{-1})
400	2.7	0.038
800	3.0	0.028

TABLE S1. **Refractive indices and absorbances of graphite.** Refractive indices n and absorbances α used to compute the absorbed fluence with Eq. (S2) for the two pump wavelengths λ, taken from (53).

$A = 1 - e^{-\alpha d}$ the absorptivity. We used a sample thickness $d = 50$ nm and the wavelength-dependent absorbances, α, and refractive indices, n, listed in Tab. S1. The experiments were performed at the same fluence.

The measurements were performed by looping over 32 delay points with a 2-second exposure (one second for I_{on} and one for I_{off}) with a total of over 10-minute exposure per time delay. One delay point represents 4.9×10^9 acquired electrons with 50% being unscattered.

The obtained diffraction patterns (see Fig. S3b) were averaged along the 6-fold symmetry axis to account for the slight zone-axis misalignment (7). We also compensated for the shift of 0.1 pixels of the zero-order peak with a linear 2D interpolation. We used a calibration constant of 0.031 Å^{-1}/pixel, such that the 512×512 pixels^2 detection window spans a 15×15 Å^{-2} area in reciprocal space.

S2. Time-resolved dynamics of graphite diffraction patterns

Figure S4a shows the photoinduced transient dynamics at different points in reciprocal space when using 3.1 eV and 1.55 eV pump pulses. The areas of interest lie in the diffuse scattering, at the K point (blue line), between the Γ and M points around the {110} Bragg peaks (magenta line), and at the family of {100} Bragg peaks (black line). Figure S4b-i shows the intensity variation of the diffraction patterns integrated for specific time delays after photoexcitation with 1.55 eV pulse energy (panels b-e) and 3.1 eV pulse energy (panels f-i). The regions of interest are highlighted by circles with the same color code as Fig. S4a. The diffuse-scattering integration regions around K and $\Gamma \to M$ span an area of 0.304 Å^{-2}, while the region around each of the Bragg peaks in the {100} family spans an area of 0.012 Å^{-2}.

The Bragg peak intensity decreases by 0.1% due to the Debye-Waller effect, while the

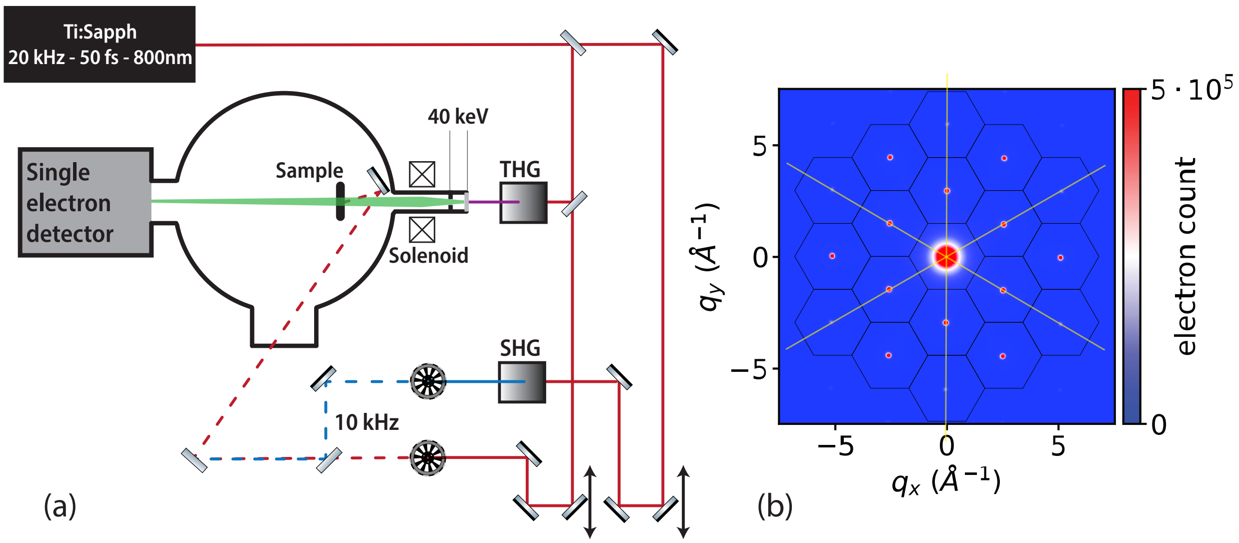

FIG. S3. **Illustration of the UED measurements.** (a) Scheme of the used UED setup. An 800 nm laser beam is divided into three beam-lines. The probe passes through the third-harmonics generator (THG) before back-illuminating the photocathode, which emits electron pulses. The two pump beam-line pulses are delayed with respect to the probe pulse through two mechanical delay stages. One of the pump beams passes through a second-harmonics generator (SHG). Two photoexcitation energies (1.55 eV, red-dashed line; and 3.1 eV, blue-dashed line) can be achieved with similar beam properties. The pump pulses are mechanically chopped and later collimated to reach a similar spot size. (b) Diffraction patterns of graphite averaged over one delay time. The yellow lines indicate the 6-fold symmetry axes employed to average the data.

intensity increases in the diffuse scattering regions. Initially, we observe a fast increase of 0.3% in the intensity at the K point of the Brillouin zone (BZ), followed by a slow decrease after 2 ps. This observation is also illustrated by the strong signature at the K points of the BZ on the normalized diffraction patterns at early time delay, $\Delta t = 1.3$ ps (Fig. S4c and g). It suggests a growth in the phonon population with momentum K, corresponding to strongly coupled optical A_1' phonons (23, 25). The electron intensity increases more slowly around the $\{110\}$ Bragg peak, between the Γ and M points of the BZ which is visible at later time delay $\Delta t = 8$ ps (Fig. S4e and i). This effect can be explained as an increase in the population of acoustic phonons with momentum spanning the $\Gamma \rightarrow K$ direction, arising from phonon-phonon scattering of high-energy optical phonons. These observations are consistent with previous ultrafast diffuse electron scattering experiments with 1.55 eV

photoexcitation (7, 22).

The diffuse scattering at the $\mathit{\Gamma}_{100}$ described in the main manuscript can be interpreted as a narrowing of the Bragg peak after photoexcitation with a 3.1 eV pump. Figure S5 shows the dynamics of the full width at half maximum (FWHM) along q_x for both photoexcitations at 1.55 eV and 3.1 eV. The Bragg peak is fitted with a two-dimensional Voigt function, whose expression reads

$$V(x,y) = \text{offset} + \text{amplitude} \cdot \left(\frac{\text{Re}\left[w\left(\frac{(q_x - q_{x_0}) + i\gamma_x}{\sigma_x \sqrt{2}} \right) \right]}{\sigma_x \sqrt{2\pi}} \right) \cdot \left(\frac{\text{Re}\left[w\left(\frac{(q_y - q_{y_0}) + i\gamma_y}{\sigma_y \sqrt{2}} \right) \right]}{\sigma_y \sqrt{2\pi}} \right). \quad \text{(S3)}$$

Where $\text{Re}(w(\cdot))$ is the real part of the complex error function. Thus the FHWM in both directions read:

$$\text{FWHM}_{x/y} = 0.5346 \cdot 2\gamma_{x/y} + \sqrt{0.2166 \cdot \left(2\gamma_{x/y}\right)^2 + \left(2\sqrt{2}\log(2)\sigma_{x/y}\right)^2} \quad \text{(S4)}$$

We fit both I_{on} and I_{off} and compute the deviation of the FWHM of the photoexcited Bragg peak from the unperturbed one, as

$$\mathit{\Delta}\text{FWHM}_{x/y} = \text{FWHM}_{x/y}^{(\text{on})} - \text{FWHM}_{x/y}^{(\text{off})}. \quad \text{(S5)}$$

From Fig. S5, we observe a decrease in the FWHM with 3.1 eV photoexcitation whereas the FWHM with 1.55 eV remains unchanged.

S3. First-principles modeling of EELS in graphite

A. DFT-based calculation of the dielectric function and EELS

The electronic structure of graphite is obtained from first principles using density functional theory (DFT) simulations with the Perdew–Burke–Ernzerhof (PBE) exchange-correlation functional as implemented in the Quantum Espresso code (45, 46). We use optimized norm-conserving Vanderbilt pseudopotentials to construct the atomic structure of graphite (54). The inter-atomic distances in graphite are set to values corresponding to the experimentally reported in-plane and out-of-plane lattice constants, **a** = **b** = 2.46 Å and **c** = 6.71 Å, respectively (55). The van der Waals forces are included in the calculation of interlayer distances using the DFT-D2 method of Grimme (56). The plane-wave energy

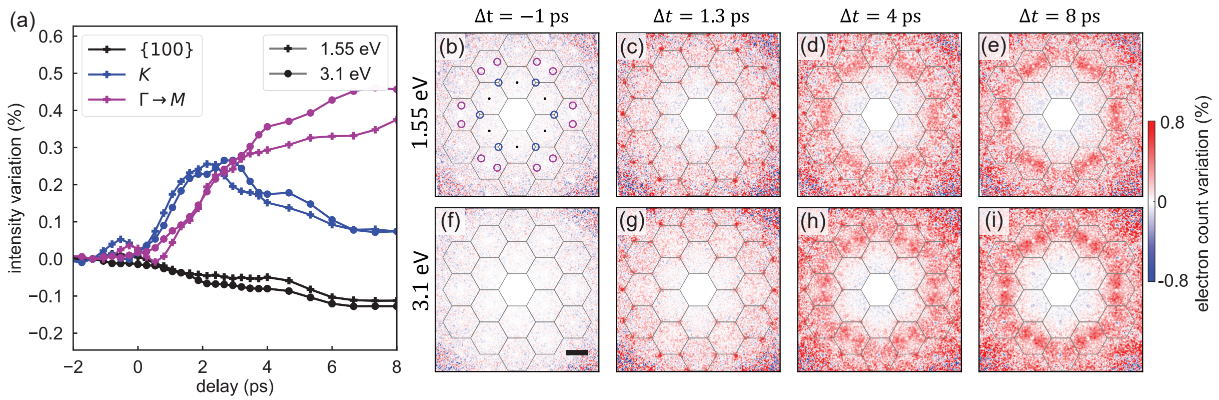

FIG. S4. **Snapshots of the UED dynamics and extracted time traces.** (a) Transient electron dynamics averaged over different regions of reciprocal space upon photoexcitation with 1.55 eV and 3.1 eV pulses (cross and round markers, respectively). The black curve corresponds to the electron count variation averaged over the {100} family of diffracted electron beams. The magenta and blue curves correspond to the electron count variation at the K point and between the Γ and M points of the reduced Brillouin zones (BZs). These selected regions of interest correspond to the circles in panel b) with the same color code. (b-e) Intensity variation resolved in momentum space at selected time delays after photoexcitation with 1.55 eV pump pulses. (f-i) Same as (b-e) with 3.1 eV pump pulses. The thick black line in panel g) corresponds to 2 Å^{-1}.

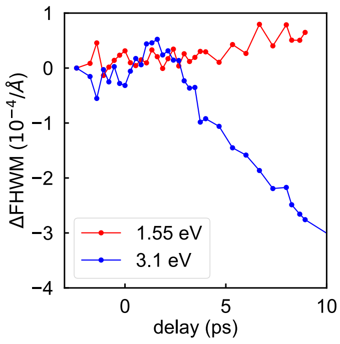

FIG. S5. **Narrowing of the Γ_{100} Bragg peak.** Dynamics of the FHWM along q_x scattered momentum for a photoexcitation with 1.55 eV (in red) and 3.1 eV (in blue) pump pulse energy.

cutoff is set to 80 Ry. The first BZ is sampled with a $32 \times 32 \times 6$ **k**-point mesh. Using the resulting Kohn-Sham wave functions and eigenenergies, the macroscopic dielectric function

is calculated within the independent-particle approximation as (47, 57, 58)

$$\epsilon_{\rm M}(\mathbf{q},\omega) = 1 - \frac{4\pi g_s e^2}{|\mathbf{q}|^2 N_{\mathbf{k}} V \hbar} \sum_{nm\mathbf{k}} \varOmega^{\mathbf{q}}_{nm\mathbf{k}} (\varOmega^{\mathbf{q}}_{nm\mathbf{k}})^* \left[\frac{f_{m\mathbf{k}}(1-f_{n\mathbf{k}-\mathbf{q}})}{\omega - \varepsilon_{m\mathbf{k}} + \varepsilon_{n\mathbf{k}-\mathbf{q}} - \mathrm{i}\gamma} - \frac{f_{m\mathbf{k}}(1-f_{n\mathbf{k}-\mathbf{q}})}{\omega + \varepsilon_{m\mathbf{k}} - \varepsilon_{n\mathbf{k}-\mathbf{q}} + \mathrm{i}\gamma} \right]. \tag{S6}$$

Here, $\mathbf{k}$ runs over the noted discrete mesh, $N_{\mathbf{k}} = 32 \times 32 \times 6$ is the number of $\mathbf{k}$ points in the first BZ, $\mathbf{q}$ is the 3D wave vector transfer, ω is the optical frequency, V is the unit-cell volume, m and n are band indexes, we take $g_s = 2$ for spin-unpolarized calculations, $f_{n\mathbf{k}}$ denotes the occupation of states $(n,\mathbf{k})$, and $\hbar\varepsilon_{n\mathbf{k}}$ stands for the corresponding Kohn-Sham eigenenergies. In order to obtain the probability associated with electron energy-loss spectroscopy (EELS), we need to calculate the in-plane $\epsilon_{\parallel}(\mathbf{q}_{\parallel},\omega)$ and out-of-plane $\epsilon_z(\omega)$ dielectric tensor components of graphite. In particular, the former, which depends on the in-plane wave vector transfer component $\mathbf{q}_{\parallel}$, is calculated using the matrix elements $\varOmega^{\mathbf{q}_{\parallel}}_{nm\mathbf{k}} = \langle n\mathbf{k}|\mathrm{e}^{\mathrm{i}\mathbf{q}_{\parallel}\cdot\mathbf{R}}|m\mathbf{k}-\mathbf{q}_{\parallel}\rangle$ as obtained from the Yambo code for a $\mathbf{q}_{\parallel}$-grid within the first BZ (47). In addition, we assume the out-of-plane dielectric function component to be local (i.e., momentum-independent) within the range of values of the z component of the transferred wave vector ω/v, where v is the electron velocity ($v \approx 0.695\,c$ for the 200 keV electrons under consideration). In fact, the out-of-plane wave vector transfer ω/v is also neglected in $\epsilon_{\parallel}$ (i.e., $q_z \approx 0$), as it is small compared to the size of the BZ. Subsequently, we calculate the EELS of graphite as a function of in-plane wave vector transfer and frequency using the following equation (48):

$$\Gamma_{\rm EELS}(\mathbf{q}_{\parallel},\omega) = \frac{e^2 L}{\pi^2\hbar v^2} \mathrm{Im}\left\{ \frac{v^2/c^2 - 1/\epsilon_{\parallel}(\mathbf{q}_{\parallel},\omega)}{q_{\parallel}^2 + \left[\epsilon_z(\omega)/\epsilon_{\parallel}(\mathbf{q}_{\parallel},\omega)\right]\left(\omega^2/v^2\right) - \epsilon_z(\omega)\omega^2/c^2} \right\}, \tag{S7}$$

where L is the length of the electron trajectory inside the bulk of graphite.

B. Atomic-level simulations of in-plane lattice dynamics in graphite

Understanding the atomic-level relaxation mechanisms of photoexcited electrons is crucial for interpreting the temporal variations observed in EELS experiments. To achieve this, we begin our analysis by simulating the E_{2g} phonon mode at the Γ point and the A_1' mode at the K point, where strong electron-phonon coupling occurs in graphite (7, 25). The left panels of Figs. S6a and S6b schematically display tilted side views of the atomic vibrations associated with the E_{2g} and A_1' optical phonons in graphite at 0.198 and 0.165 eV energies,

respectively (25). These modes result from the opposing motion of atoms in carbon dimers along their bond directions within the hexagonal plane. In the E_{2g} mode, these dimers are orientated parallel (59, 60), and the unit cell can be modeled with four atoms as shown in the right-top panel of Fig. S6a. In contrast, in the A_1' mode, the dimers are attached to each carbon atom in the central hexagon and vibrate toward the center of the hexagon, and consequently, to simulate this mode we consider a larger, twelve-atom unit cell, as illustrated in the upper-right panel of Fig. S6b. We then determine the EELS probability for a grid of $\mathbf{q}_\parallel$ points by averaging the computed values along the $\mathbf{q}_1$ and $\mathbf{q}_2$ directions (which correspond to bond directions in real space) within the relevant BZs for the E_{2g} and A_1' modes, as shown in the lower-right panels of Figs. S6a and S6b, using the expression

$$\Gamma_{\rm EELS}(\mathbf{q}_\parallel,\omega)=\frac{1}{3}\Big[\Gamma_{\rm EELS}(\mathbf{q}_1,\omega)+2\Gamma_{\rm EELS}(\mathbf{q}_2,\omega)\Big], \quad \text{(S8)}$$

where the average over different C-C bond orientations results in the factors of one along the $\mathbf{q}_1$ direction and two along the $\mathbf{q}_2$ direction, which are here incorporated because we are considering a representative in-plane bond. This calculation method, which is computationally feasible using a medium-size computer cluster, should yield a reasonable approximation to the full BZ average, which becomes computationally prohibitive.

C. Changes produced in the EELS probability by thermal excitation of in-plane phonon modes

Here, we simulate the variation in the EELS probability due to vibrations corresponding to the E_{2g} and A_1' optical phonon modes. Noticing that these modes are approximately dispersionless near the Γ and K points, we estimate the change in the EELS probability by calculating the permittivity using Eq. (S6) for various bond displacements x around the equilibrium C-C bond distance d. The displacements are equally repeated in all unit cells, as corresponding to the Γ point. The resulting EELS probability $\Gamma_{d+x}(\mathbf{q}_\parallel,\omega)$ (see Sec. S3 B) is then dependent on x. The end result for the thermal average of the EELS probability is obtained from these calculations as

$$\Gamma^T(\mathbf{q}_\parallel,\omega)=\int dx\;\Gamma_{d+x}(\mathbf{q}_\parallel,\omega)P(x,T), \quad \text{(S9)}$$

where we introduce a weight corresponding to the temperature-dependent probability $P(x,T)$ associated with each displacement x. The latter is obtained from a simple one-

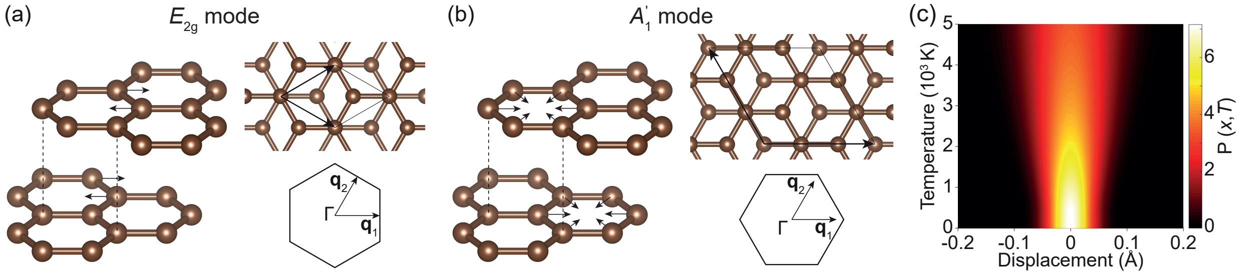

FIG. S6. **Representation of the phonon displacements considered in the calculations.** (a) Tilted side view of the atomic displacements associated with the phonon mode E_{2g} (represented by black arrows in the left sketches), along with a top view of the unit cell (upper-right sketch), and the Brillouin zone (lower-right scheme) of the corresponding real-space unit cell. Transferred wave-vector directions are shown by $\mathbf{q}_1$ and $\mathbf{q}_2$ in the Brillouin zone. (b) Same as (a), but for the A_1' phonon mode. (c) Probability distribution $P(x,T)$ of the C-C distance as a function of thermal displacement x and temperature T. The integral of $P(x,T)$ over x is normalized to unity. The probability is obtained from a 1D quantum-harmonic oscillator model with parameters associated with the in-plane optical phonon modes of graphite.

dimensional quantum harmonic oscillator, as explained below.

In practice, we approximate Eq. (S9) as

$$\Gamma^T(\mathbf{q}_\parallel,\omega) \approx \sum_{j=0}^{N-1} \Gamma_{d+x_j}(\mathbf{q}_\parallel,\omega) \int_{x_j-h/2}^{x_j+h/2} dx \; \mathrm{P}(x,T), \tag{S10}$$

where we use a discrete grid formed by N displacements x_j equally spaced by a step h and spanning a region centered around $x=0$ (see below). Also, the x-dependent probability is considered to be nearly constant within each displacement element of size h.

The probability $P(x,T)$ is obtained by considering the wave functions of the quantum harmonic oscillator,

$$\psi_n(x) = \frac{1}{\sqrt{2^n n!}}\left(\frac{M\omega}{\pi\hbar}\right)^{1/4} \exp\left\{-\frac{M\omega x^2}{2\hbar}\right\} H_n\left(\sqrt{\frac{M\omega}{\hbar}}x\right), \tag{S11}$$

where H_n is a Hermite polynomial and $M = M_\mathrm{C}/2$ is the reduced mass corresponding the C-C dimer with M_C denoting the carbon atomic mass. Using the Boltzmann distribution function, $p_n(T) = \mathrm{e}^{-\frac{n\hbar\omega}{k_\mathrm{B}T}}(1-\mathrm{e}^{-\frac{\hbar\omega}{k_\mathrm{B}T}})$, for the occupation of state n, we obtain

$$P(x,T) = \sum_n |\psi_n(x)|^2 \, p_n(T). \tag{S12}$$

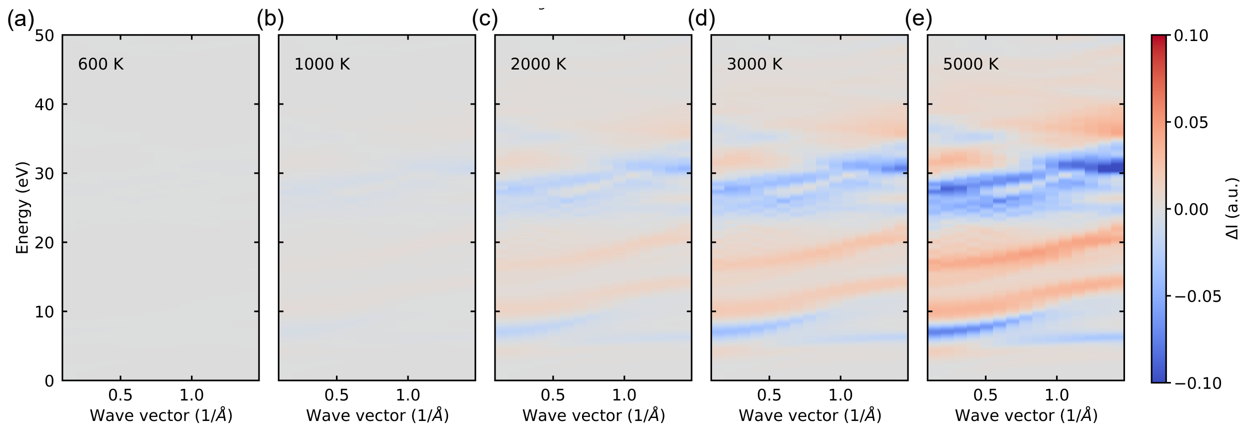

FIG. S7. **Electron energy loss spectra calculated variation with E_{2g} phonons.** (a)-(e) Change in the graphite EELS probability as a function of momentum produced by the thermal population of the E_{2g} optical phonon mode at temperatures of 600, 1000, 2000, 3000, and 5000 K, respectively. The equilibrium temperature for computing the variation is 300 K.

We set $\hbar\omega = 0.198$ and 0.165 eV to represent in-plane E_{2g} and A_1' modes, respectively. The resulting distribution $P(x,T)$ for the E_{2g} mode is plotted in Fig. S6c over a wide temperature range.

As expected, $P(x,T)$ decreases rapidly for atomic displacements larger than ~ 0.1 Å, and therefore, we find it safe to evaluate Eq. (S10) using $N = 19$ points x_j in the ± 0.18 Å range (i.e., with $h = 0.02$ Å). In addition, the in-plane momentum transfer $\mathbf{q}_\parallel$ used in our calculations is directly determined by the $\mathbf{k}$-point grid, with a minimum $|\mathbf{q}_\parallel|$ of ~ 0.09 Å^{-1}.

To illustrate the effect of the E_{2g} and A_1' modes on the graphite EELS probability, we calculate the variation relative to room temperature (RT) when we move to a mode temperature T of a few 1000's K, that is, $\Delta\Gamma(\mathbf{q}_\parallel,\omega) = \Gamma^T(\mathbf{q}_\parallel,\omega) - \Gamma^{RT}(\mathbf{q}_\parallel,\omega)$. As shown in the results plotted in Fig. S7 and Fig. S8, while the low-energy-loss regions of the calculated maps show similar variations as in the experiment (see Fig. 4b in the main text), the variations at high energies differ from experiments.

D. The effect of lattice thermal expansion on the EELS probability in graphite

As an additional mechanism to explain the experimental observations, we investigate the effect of pump-induced lattice thermal expansion on the EELS maps of graphite. To this end, we incorporate the thermal expansion produced in the inter-atomic bond distances and the

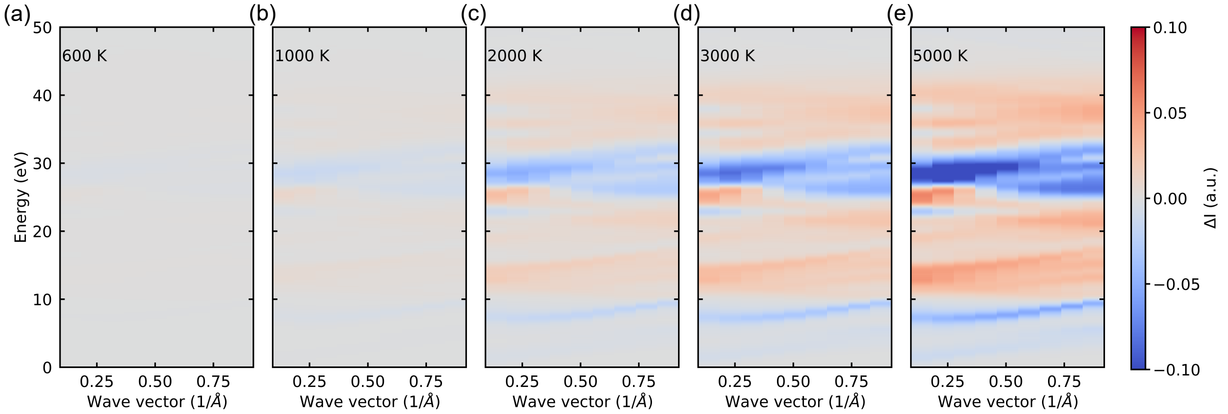

FIG. S8. **Electron energy loss spectra calculated variation with A_1' phonons.** (a)-(e) Change in the graphite EELS probability as a function of momentum produced by the thermal population of the A_1' optical phonon mode at temperatures of 600, 1000, 2000, 3000, and 5000 K, respectively. The equilibrium temperature for computing the variation is 300 K.

inter-layer separations. The temperature-dependent lattice dynamics of graphite has been well-studied theoretically and experimentally (32, 61). In particular, the in-plane, $\alpha_a(T)$, and out-of-plane, $\alpha_c(T)$, linear thermal expansion coefficients are reported in a previous study (33), defined as a function of temperature as $\alpha(T) = L^{-1}(dL/dT)$, where L is the length of the specimen along the considered crystallographic direction (a or c). We linearly interpolate the measured thermal expansion coefficients as shown in Fig. S9a. The changes in the in-plane and out-plane lattice parameters relative to RT are then given by $\Delta a = a\int_{RT}^{T_L} dT'\ \alpha_a(T')$ and $\Delta c = c\int_{RT}^{T_L} dT'\ \alpha_c(T')$, respectively. Here, T_L is the lattice expansion temperature, which depends on pump fluence and time delay through the cascade of heat flow from the incident light to the electronic system, and from here to optical and acoustic phonons. Employing this method, we obtain expanded lattice properties of graphite for $T_L = 600,\ 1000,\ 1500,$ and 2000 K, and then we calculate EELS maps for the corresponding atomic configurations generated for each temperature (i.e., including thermal expansion). In Fig. S9b-e, we show the variation of the EELS map obtained for these temperatures with respect to the EELS map at room temperature, that is, $\Delta\Gamma(\mathbf{q}_\parallel,\omega) = \Gamma^{T_L}(\mathbf{q}_\parallel,\omega) - \Gamma^{RT}(\mathbf{q}_\parallel,\omega)$. The magnitude of the change in the EELS probability due to thermal expansion is quite consistent with the values observed in experiments. However, the variations observed in the low- and high-energy-loss regions of the calculated EELS maps clearly indicate that the pump-induced effects observed in our experiments cannot be explained by lattice expansion alone.

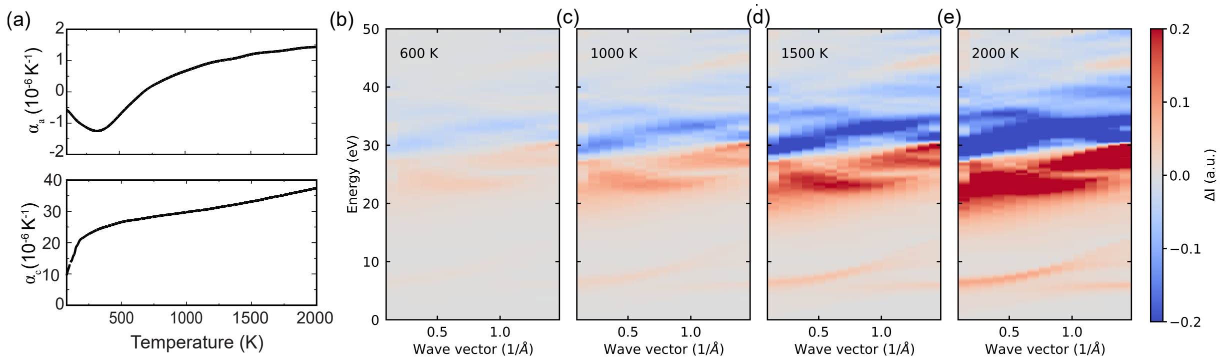

FIG. S9. **Electron energy loss spectra calculated variation with out-of-plane thermal expansion.** (a) In-plane (top panel) and out-of-plane (bottom panel) thermal expansion coefficients of graphite as a function of temperature, calculated by interpolating experimental data from Refs. (32, 33). (b)-(e) Variation of the graphite EELS probability due to temperature-induced lattice expansion for temperatures of 600, 1000, 1500, and 2000 K, respectively. We consider an equilibrium temperature of 300 K.

Photon energy (eV)	T$_{A_1'}$ (K)	T$_{E_{2g}}$ (K)	T$_L$ (K)
1.55	7×10^2	9×10^2	1×10^3
3.1	2.5×10^3	5×10^3	4×10^2

TABLE S2. **Fitting parameters for two pump energies.** Parameters resulting from a fit of the simulations to the experimental data.

E. Combined results and discussion

We conclude that the combination of pump-induced lattice expansion and strong coupling of photo-excited electrons to optical phonons seems to be the most likely scenario explaining the changes in the EELS maps observed in experiments. To validate this hypothesis, utilizing the listed values in Tab. S2 for the E_{2g} and A_1' phonon temperatures ($T_{E_{2g}}$ and $T_{A_1'}$) and lattice expansion temperature (T_L), along with the fitting weights provided in the main text ($w_{A_1'} = w_{E_{2g}} = 0.25$, $w_L = 0.5$), we obtained the EELS variations shown in Figure 4. Our simulations accurately explain the measurements for the 3.1 eV-pump-induced variations in the EELS map. However, the experimentally observed map for 1.55 eV pumping exhibits notable discrepancies in the low wave vector transfer region.

Caption for Movie S1. **Side by side diffuse scattering for a photoexcitation with 3.1 eV and 1.55 eV.** Normalized diffraction patterns along the time delay for a photoexcitation with 3.1 eV (left) and 1.55 eV (right).

REFERENCES AND NOTES

1. K. S. Novoselov, A. Mishchenko, A. Carvalho, A. H. C. Neto, 2D materials and van der Waals heterostructures. *Science* **353**, aac9439 (2016).

2. Y. Cao, V. Fatemi, S. Fang, K. Watanabe, T. Taniguchi, E. Kaxiras, P. Jarillo-Herrero, Unconventional superconductivity in magic-angle graphene superlattices. *Nature* **556**, 43–50 (2018).

3. M. V. Stern, Y. Waschitz, W. Cao, I. Nevo, K. Watanabe, T. Taniguchi, E. Sela, M. Urbakh, O. Hod, M. B. Shalom, Interfacial ferroelectricity by van der Waals sliding. *Science* **372**, 1462–1466 (2021).

4. K. F. Mak, K. He, J. Shan, T. F. Heinz, Control of valley polarization in monolayer MoS_2 by optical helicity. *Nat. Nanotechnol.* **7**, 494–498 (2012).

5. J. R. Schaibley, H. Yu, G. Clark, P. Rivera, J. S. Ross, K. L. Seyler, W. Yao, X. Xu, Valleytronics in 2D materials. *Nat. Rev. Mater.* **1**, 16055 (2016).

6. A. Rycerz, J. Tworzydło, C. W. J. Beenakker, Valley filter and valley valve in graphene. *Nat. Phys.* **3**, 172–175 (2007).

7. M. J. Stern, L. P. René de Cotret, M. R. Otto, R. P. Chatelain, J.-P. Boisvert, M. Sutton, B. J. Siwick, Mapping momentum-dependent electron-phonon coupling and nonequilibrium phonon dynamics with ultrafast electron diffuse scattering. *Phys. Rev. B.* **97**, 165416 (2018).

8. J. Madéo, M. K. L. Man, C. Sahoo, M. Campbell, V. Pareek, E. L. Wong, A. Al-Mahboob, N. S. Chan, A. Karmakar, B. M. K. Mariserla, X. Li, T. F. Heinz, T. Cao, K. M. Dani, Directly visualizing the momentum-forbidden dark excitons and their dynamics in atomically thin semiconductors. *Science* **370**, 1199–1204 (2020).

9. M. P. M. Dean, Y. Cao, X. Liu, S. Wall, D. Zhu, R. Mankowsky, V. Thampy, X. M. Chen, J. G. Vale, D. Casa, J. Kim, A. H. Said, P. Juhas, R. Alonso-Mori, J. M. Glownia, A. Robert, J. Robinson, M. Sikorski, S. Song, M. Kozina, H. Lemke, L. Patthey, S. Owada, T. Katayama, M. Yabashi, Y. Tanaka, T. Togashi, J. Liu, C. Rayan Serrao, B. J. Kim, L. Huber, C. L. Chang,

D. F. McMorrow, M. Först, J. P. Hill, Ultrafast energy- and momentum-resolved dynamics of magnetic correlations in the photo-doped Mott insulator Sr_2IrO_4. *Nat. Mater.* **15**, 601–605 (2016).

10. M. Mitrano, S. Lee, A. A. Husain, L. Delacretaz, M. Zhu, G. de la Peña Munoz, S. X.-L. Sun, Y. I. Joe, A. H. Reid, S. F. Wandel, G. Coslovich, W. Schlotter, T. van Driel, J. Schneeloch, G. D. Gu, S. Hartnoll, N. Goldenfeld, P. Abbamonte, Ultrafast time-resolved x-ray scattering reveals diffusive charge order dynamics in $La_{2-x}Ba_xCuO_4$. *Sci. Adv.* **5**, eaax3346 (2019).

11. S. Wandel, F. Boschini, E. H. da Silva Neto, L. Shen, M. X. Na, S. Zohar, Y. Wang, S. B. Welch, M. H. Seaberg, J. D. Koralek, G. L. Dakovski, W. Hettel, M.-F. Lin, S. P. Moeller, W. F. Schlotter, A. H. Reid, M. P. Minitti, T. Boyle, F. He, R. Sutarto, R. Liang, D. Bonn, W. Hardy, R. A. Kaindl, D. G. Hawthorn, J.-S. Lee, A. F. Kemper, A. Damascelli, C. Giannetti, J. J. Turner, G. Coslovich, Enhanced charge density wave coherence in a light-quenched, high-temperature superconductor. *Science* **376**, 860–864 (2022).

12. G. Moos, C. Gahl, R. Fasel, M. Wolf, T. Hertel, Anisotropy of quasiparticle lifetimes and the role of disorder in graphite from ultrafast time-resolved photoemission spectroscopy. *Phys. Rev. Lett.* **87**, 267402 (2001).

13. T. Kampfrath, L. Perfetti, F. Schapper, C. Frischkorn, M. Wolf, Strongly coupled optical phonons in the ultrafast dynamics of the electronic energy and current relaxation in graphite. *Phys. Rev. Lett.* **95**, 187403 (2005).

14. F. Carbone, O. H. Kwon, A. H. Zewail, Dynamics of chemical bonding mapped by energy-resolved 4D electron microscopy. *Science* **325**, 181–184 (2009).

15. S. Pagliara, G. Galimberti, S. Mor, M. Montagnese, G. Ferrini, M. S. Grandi, P. Galinetto, F. Parmigiani, Photoinduced π-π* band gap renormalization in graphite. *J. Am. Chem. Soc.* **133**, 6318–6322 (2011).

16. M. Breusing, C. Ropers, T. Elsaesser, Ultrafast carrier dynamics in graphite. *Phys. Rev. Lett.* **102**, 086809 (2009).

17. F. Carbone, P. Baum, P. Rudolf, A. H. Zewail, Structural preablation dynamics of graphite observed by ultrafast electron crystallography. *Phys. Rev. Lett.* **100**, 035501 (2008).

18. R. P. Chatelain, V. R. Morrison, B. L. M. Klarenaar, B. J. Siwick, Coherent and incoherent electron-phonon coupling in graphite observed with radio-frequency compressed ultrafast electron diffraction. *Phys. Rev. Lett.* **113**, 235502 (2014).

19. F. Carbone, B. Barwick, O. H. Kwon, H. S. Park, J. S. Baskin, A. H. Zewail, EELS femtosecond resolved in 4D ultrafast electron microscopy. *Chem. Phys. Lett.* **468**, 107–111 (2009).

20. R. M. van der Veen, T. J. Penfold, A. H. Zewail, Ultrafast core-loss spectroscopy in four-dimensional electron microscopy. *Struct. Dyn.* **2**, 024302 (2015).

21. L. P. de René Cotret, J.-H. Pöhls, M. J. Stern, M. R. Otto, M. Sutton, B. J. Siwick, Time- and momentum-resolved phonon population dynamics with ultrafast electron diffuse scattering. *Phys. Rev. B.* **100**, 214115 (2019).

22. A. Stange, C. Sohrt, L. X. Yang, G. Rohde, K. Janssen, P. Hein, L.-P. Oloff, K. Hanff, K. Rossnagel, M. Bauer, Hot electron cooling in graphite: Supercollision versus hot phonon decay. *Phys. Rev. B.* **92**, 184303 (2015).

23. J.-A. Yang, S. Parham, D. Dessau, D. Reznik, Novel electron-phonon relaxation pathway in graphite revealed by time-resolved raman scattering and angle-resolved photoemission spectroscopy. *Sci. Rep.* **7**, 40876 (2017).

24. M. X. Na, A. K. Mills, F. Boschini, M. Michiardi, B. Nosarzewski, R. P. Day, E. Razzoli, A. Sheyerman, M. Schneider, G. Levy, S. Zhdanovich, T. P. Devereaux, A. F. Kemper, D. J. Jones, A. Damascelli, Direct determination of mode-projected electron-phonon coupling in the time domain. *Science* **366**, 1231–1236 (2019).

25. H. Beyer, P. Hein, K. Rossnagel, M. Bauer, Ultrafast decay of carrier momentum anisotropy in graphite. *Phys. Rev. B.* **107**, 115136 (2023).

26. F. H. L. Koppens, D. E. Chang, F. J. García de Abajo, Graphene plasmonics: A platform for strong light-matter interactions. *Nano Lett.* **11**, 3370–3377 (2011).

27. A. N. Grigorenko, M. Polini, K. S. Novoselov, Graphene plasmonics. *Nat. Photon.* **6**, 749–758 (2012).

28. R. Claude, M. Puppin, B. Weaver, P. Usai, T. LaGrange, F. Carbone, Shot-to-shot acquisition ultrafast electron diffraction. arXiv:2502.02540v1 [physics.ins-det] (2025).

29. S. C. Liou, C.-S. Shie, C. H. Chen, R. Breitwieser, W. W. Pai, G. Y. Guo, M.-W. Chu, π-Plasmon dispersion in free-standing graphene by momentum-resolved electron energy-loss spectroscopy. *Phys. Rev. B* **91**, 045418 (2015).

30. A. G. Marinopoulos, L. Reining, A. Rubio, V. Olevano, Ab initio study of the optical absorption and wave-vector-dependent dielectric response of graphite. *Phys. Rev. B.* **69**, 245419 (2004).

31. N. Mounet, N. Marzari, First-principles determination of the structural, vibrational and thermodynamic properties of diamond, graphite, and derivatives. *Phys. Rev. B.* **71**, 205214 (2005).

32. H. O. Pierson, *Handbook of Carbon, Graphite, Diamonds and Fullerenes: Processing, Properties and Applications* (William Andrew Park Ridge, 1993).

33. A. B. Kuzmenko, L. Benfatto, E. Cappelluti, I. Crassee, D. van der Marel, P. Blake, K. S. Novoselov, A. K. Geim, Gate tunable infrared phonon anomalies in bilayer graphene. *Phys. Rev. Lett.* **103**, 116804 (2009).

34. J. D. Perkins, J. M. Graybeal, M. A. Kastner, R. J. Birgeneau, J. P. Falck, M. Greven, Mid-infrared optical absorption in undoped lamellar copper oxides. *Phys. Rev. Lett.* **71**, 1621–1624 (1993).

35. J. Lorenzana, G. A. Sawatzky, Phonon assisted multimagnon optical absorption and long lived two-magnon states in undoped lamellar copper oxides. *Phys. Rev. Lett.* **74**, 1867–1870 (1995).

36. J. Levallois, M. K. Tran, D. Pouliot, C. N. Presura, L. H. Greene, J. N. Eckstein, J. Uccelli, E. Giannini, G. D. Gu, A. J. Leggett, D. van der Marel, Temperature-dependent ellipsometry measurements of partial coulomb energy in superconducting cuprates. *Phys. Rev. X.* **6**, 031027 (2016).

37. M. Mitrano, A. A. Husain, S. Vig, A. Kogar, M. S. Rak, S. I. Rubeck, J. Schmalian, B. Uchoa, J. Schneeloch, R. Zhong, G. D. Gu, P. Abbamonte, Anomalous density fluctuations in a strange metal. *Proc. Natl. Acad. Sci. U.S.A.* **115**, 5392–5396 (2018).

38. F. Barantani, M. K. Tran, I. Madan, I. Kapon, N. Bachar, T. C. Asmara, E. Paris, Y. Tseng, W. Zhang, Y. Hu, E. Giannini, G. Gu, T. P. Devereaux, C. Berthod, F. Carbone, T. Schmitt, D. van der Marel, Resonant inelastic x-ray scattering study of electron-exciton coupling in high-T_c cuprates. *Phys. Rev. X* **12**, 021068 (2022).

39. D. Fausti, R. I. Tobey, N. Dean, S. Kaiser, A. Dienst, M. C. Hoffmann, S. Pyon, T. Takayama, H. Takagi, A. Cavalleri, Light-induced superconductivity in a stripe-ordered cuprate. *Science* **331**, 189–191 (2011).

40. X. Li, T. Qiu, J. Zhang, E. Baldini, J. Lu, A. M. Rappe, K. A. Nelson, Terahertz field-induced ferroelectricity in quantum paraelectric $SrTiO_3$. *Science* **364**, 1079–1082 (2019).

41. T. Nova, A. Disa, M. Fechner, A. Cavalleri, Metastable ferroelectricity in optically strained $SrTiO_3$. *Science* **364**, 1075–1079 (2018).

42. L. Piazza, M. Cottet, F. Carbone, D. Masiel, T. LaGrange, Principles and implementation of an ultrafast transmission electron microscope. *Microsc. Microanal.* **18**, 600–601 (2012).

43. L. Piazza, D. Masiel, T. LaGrange, B. Reed, B. Barwick, F. Carbone, Design and implementation of a fs-resolved transmission electron microscope based on thermionic gun technology. *Chem. Phys.* **423**, 79–84 (2013).

44. J. P. Perdew, K. Burke, M. Ernzerhof, Generalized gradient approximation made simple. *Phys. Rev. Lett.* **77**, 3865–3868 (1996).

45. P. Giannozzi, S. Baroni, N. Bonini, M. Calandra, R. Car, C. Cavazzoni, D. Ceresoli, G. L. Chiarotti, M. Cococcioni, I. Dabo, A. D. Corso, S. de Gironcoli, S. Fabris, G. Fratesi, R. Gebauer, U. Gerstmann, C. Gougoussis, A. Kokalj, M. Lazzeri, L. Martin-Samos, N. Marzari, F. Mauri, R. Mazzarello, S. Paolini, A. Pasquarello, L. Paulatto, C. Sbraccia, S. Scandolo, G. Sclauzero, A. P. Seitsonen, A. Smogunov, P. Umari, R. M. Wentzcovitch, QUANTUM ESPRESSO: A modular and open-source software project for quantum simulations of materials. *J. Phys. Condens. Matter* **21**, 395502 (2009).

46. D. Sangalli, A. Ferretti, H. Miranda, C. Attaccalite, I. Marri, E. Cannuccia, P. Melo, M. Marsili, F. Paleari, A. Marrazzo, G. Prandini, P. Bonfa, M. O. Atambo, F. Affinito, M. Palummo, A. Molina-Sanchez, C. Hogan, M. Grüning, D. Varsano, A. Marini, Many-body perturbation theory calculations using the yambo code. *J. Phys. Condens. Matter* **31**, 325902 (2019).

47. F. J. García de Abajo, Optical excitations in electron microscopy. *Rev. Mod. Phys.* **82**, 209–275 (2010).

48. F. Barantani, R. Claude , F. Iyikanat, I. Madan, A. A. Sapozhnik, M. Puppin, B. Weaver, T. LaGrange, F. J. G. de Abajo, F. Carbone, Dataset of "Ultrafast momentum-resolved visualization of the interplay between phonon-mediated scattering and plasmons in graphite." Zenodo (2025); https://doi.org/10.5281/zenodo.14760926.

49. J. C. Slonczewski, P. R. Weiss, Band structure of graphite. *Phys. Rev.* **109**, 272–279 (1958).

50. M. Dapor, L. Calliari, M. Filippi, Computational and experimental study of π and $\pi + \sigma$ plasmon loss spectra for low energy ($\leq$1000 eV) electrons impinging on highly oriented pyrolitic graphite (HOPG). *Nucl. Instrum. Methods Phys. Res., Sect. B* **255**, 276–280 (2007).

51. G. F. Mancini, B. Mansart, S. Pagano, B. van der Geer, M. de Loos, F. Carbone, Design and implementation of a flexible beamline for fs electron diffraction experiments. *Nucl. Instrum. Methods Phys. Res., Sect. A* **691**, 113–122 (2012).

52. B. Barwick, D. J. Flannigan, A. H. Zewail, Photon-induced near-field electron microscopy. *Nature* **462**, 902–906 (2009).

53. A. B. Djurišić, E. H. Li, Optical properties of graphite. *J. Appl. Phys.* **85**, 7404–7410 (1999).

54. D. R. Hamann, Optimized norm-conserving vanderbilt pseudopotentials. *Phys. Rev. B.* **88**, 085117 (2013).

55. D. D. L. Chung, Review graphite. *J. Mater. Sci.* **37**, 1475–1489 (2002).

56. S. Grimme, Semiempirical GGA-type density functional constructed with a long-range dispersion correction. *J. Comput. Chem.* **27**, 1787–1799 (2006).

57. H. Ehrenreich, M. H. Cohen, Self-consistent field approach to the many-electron problem. *Phys. Rev.* **115**, 786–790 (1959).

58. S. L. Adler, Quantum theory of the dielectric constant in real solids. *Phys. Rev.* **126**, 413–420 (1962).

59. A. C. Ferrari, D. M. Basko, Raman spectroscopy as a versatile tool for studying the properties of graphene. *Nat. Nanotechnol.* **8**, 235–246 (2013).

60. M. A. Pimenta, E. del Corro, B. R. Carvalho, C. Fantini, L. M. Malard, Comparative study of Raman spectroscopy in graphene and MoS_2-type transition metal dichalcogenides. *Acc. Chem. Res.* **48**, 41–47 (2014).

61. A. C. Bailey, B. Yates, Anisotropic thermal expansion of pyrolytic graphite at low temperatures. *J. Appl. Phys.* **41**, 5088–5091 (1970).
